# Characterization of a Novel Hypotrich Ciliate From Heavy Metal-Contaminated Industrial Outlet in Onsan, Ulsan, South Korea

**DOI:** 10.3389/fmicb.2021.761961

**Published:** 2021-11-19

**Authors:** Santosh Kumar, Daizy Bharti, Shahed Uddin Ahmed Shazib, Mann Kyoon Shin

**Affiliations:** ^1^Department of Biological Sciences, College of Natural Sciences, University of Ulsan, Ulsan, South Korea; ^2^Zoological Survey of India, Kolkata, India

**Keywords:** contaminated waters, *Histriculus*, morphology, morphogenesis, phylogeny, rRNA gene

## Abstract

Very few studies exist on the description of protozoan ciliates from industrially contaminated sites. In this study, we report a description of a novel hypotrich ciliate isolated from water samples collected from an industrially contaminated outlet in Onsan, Ulsan, South Korea. The oxytrichid ciliate, *Histriculus tolerans* n. sp., was investigated using live observation and protargol impregnation. The morphology, morphogenesis, and molecular phylogeny inferred from small-subunit (SSU) rRNA gene sequences were studied. The new species is mainly characterized by a cell size of about 70 × 40 μm *in vivo*, two elongate ellipsoidal macronuclear nodules and one or two micronuclei, adoral zone of about 51% of body length with 32 membranelles on average, about 34 cirri in the right and 24 cirri in the left marginal row, 18 frontoventral transverse cirri, six dorsal kineties including two dorsomarginal rows, and dorsal kinety 1 with 26 bristles. Morphogenesis is similar to that of the type species, i.e., *Histriculus histrio*, except that oral primordium does not contribute to anlage II of the proter. Phylogenetic analyses, based on small-subunit rRNA gene sequences, consistently place the new species within the family Oxytrichidae, clustering with *H. histrio*.

## Introduction

The genus *Histriculus* Corliss, 1960, is largely confined to freshwater and marine habitats with type species, i.e., *Histriculus histrio*, having a cosmopolitan distribution. The genus is mainly characterized by undulating membranes in *Oxytricha* pattern, one right and one left marginal cirral row, confluent marginal rows at the posterior end, six dorsal kineties, and absence of caudal cirri. Berger (1999), in his detailed revision of the oxytrichids, assigned six species to the genus; however, detailed morphology and morphogenesis, as well as gene sequences, have been reported mainly for the type species, i.e., *H. histrio* (Müller, 1773), Corliss, 1960. The morphology, though poorly described, and morphogenesis with endogenous bud formation have been reported for *Histriculus vorax* (Stokes, 1891) Corliss, 1960. Berger (1999) recommended detailed reinvestigations of the remaining species of the genus.

Limited studies have been performed on the diversity of ciliates inhabiting industrially contaminated soil and water (Bharti et al., 2015). Recently, Kumar et al. (2017) discovered a novel soil ciliate isolated from contaminated soil of the petroleum industry. The present paper describes a new species of the genus *Histriculus* isolated from the heavy metal-contaminated waters, collected from the industrial outlet in Onsan, Ulsan, South Korea. A detailed description of the morphology, morphogenesis, and phylogenetic analyses based on the SSU rRNA gene sequences of the new species, i.e., *H. tolerans*, has been presented.

## Materials and Methods

### Sampling and Sample Processing

Water samples were collected on April 8, 2016 from the outlet of an industrial company in Onsan, Ulsan, South Korea (35°25′55.9′′ N 129°21′07.2′′ E). Ciliates were isolated and fed with green algae *Chlorogonium elongatum* as a food organism (Ammermann et al., 1974). Live observations were made using a microscope with differential interference contrast illuminations at a magnification range of × 100–1,000. The protargol staining method described by Kamra and Sapra (1990) was used, with some modifications, to reveal the ciliature. Measurements of impregnated specimens were performed at a magnification of × 1,000 using an ocular micrometer. A Zeiss microscope camera was employed for photomicrography. The illustration of the live specimen was prepared using free-hand sketches, while those of impregnated specimens were made with a drawing device. The terminology is according to Wallengren (1900) and Berger (1999).

### DNA Extraction, PCR Amplification, and Sequencing

Four clonal cultures were established, and the same were used for live observation and protargol impregnation to study morphology and morphogenesis. Three cells of *H. tolerans* were isolated and collected in 1.5-mL tubes for DNA extraction from a clonal culture with the help of glass micropipettes. Thus, the morphology, morphogenesis, and molecular phylogeny reported in the present study deal with the same species. The cells were washed at least three times with autoclaved distilled water for genomic DNA extraction using the REDExtract-N-Amp Tissue PCR Kit (Sigma, St. Louis, MO), following the instruction of the manufacturer, except for the reduction of each reaction volume to one-tenth (Gong et al., 2007). Amplifications of the extracted DNA were carried out using the TaKaRa ExTaq DNA polymerase Kit (TaKaRa Bio-medicals, Otsu, Japan) using the universal eukaryotic primers Euk A (FW 5′-AAC CTG GTT GAT CCT GCC AG-3′) and Euk B (RV 5′-CAC TTG GAC GTC TTC CTA GT-3′) (Medlin et al., 1988). The PCR program for SSU rRNA gene amplification included an initial denaturation at 94°C for 3 min, followed by 35 cycles of 94°C for 1 min, 56°C for 45 s, and 72°C for 80 s, with a final extension step at 72°C for 10 min. After confirmation of the appropriate size, the purified PCR products were directly sequenced on both strands on an ABI 3730 automatic sequencer at the Cosmo Genetech, Seoul, South Korea.

### Phylogenetic Analyses

For phylogenetic analyses, the SSU rRNA gene sequence of *H. tolerans* n. sp. was aligned with 54 SSU rRNA gene sequences of hypotrich ciliates from GenBank using the MAFFT software v. 7^1^ and choosing the iterative refinement methods Q-INS-I that consider the secondary structure of the SSU rRNA molecules (Katoh and Standley, 2013).

Ambiguously aligned regions were identified and excluded from the phylogenetic analyses with Gblocks v. 0.91b (Castresana, 2000) using parameters optimized for rRNA gene alignments, leaving 1,562 unambiguous positions. Maximum likelihood (ML) analyses were carried out using RAxML-HPC2 v. 8.0.24 (Stamatakis, 2014) on the CIPRES Science Gateway (Miller et al., 2010) with bootstrapping of 1,000 replicates. A Bayesian inference (BI) analysis was performed using Mr. Bayes v. 3.2.1 (Ronquist et al., 2012) and the TIM2 + I + G model, as selected by the jModel test v. 2.1.3 software (Posada, 2008) under the Akaike Information Criterion. Markov chain Monte Carlo simulations were run, with two sets of four chains using the default settings, for 1,000,000 generations with trees sampled every 100 generations and discarding the first 25% of the sampled trees as burn-in. The remaining trees were used to generate a consensus tree and to calculate the posterior probabilities of all branches using the majority rule consensus approach. Phylogenetic trees were visualized using the free software package FigTree v. 1.4 by A. Rambaut.^2^

## Results

### ZooBank Registration

The present work is registered in ZooBank under urn:lsid:zoobank.org:pub:DC0F5E57-1A3A-42E0-B24F-7B36D325A001. The new species is registered in ZooBank under urn:lsid:zoobank.org:act:EB0BB241-1F83-416D-8B16-85A18304A34A.

**FIGURE 1:**
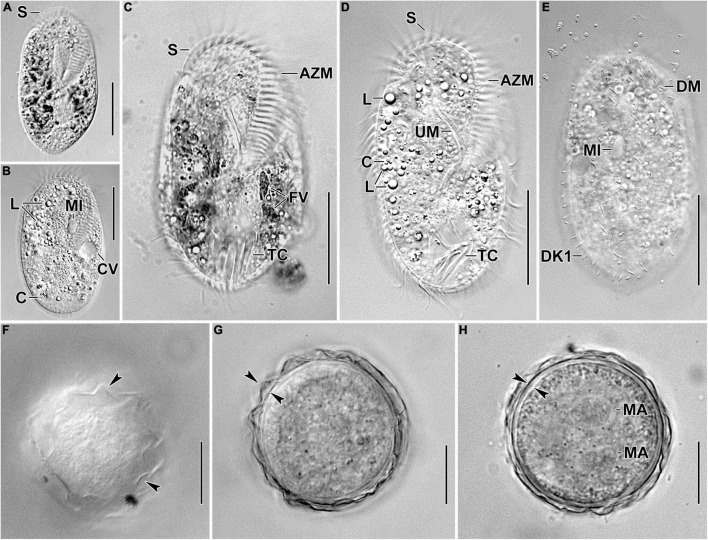
Photomicrographs of *Histriculus tolerans* n. sp. from life. **(A–D)** Ventral views of freely motile specimens, showing the body shape, the position of the contractile vacuole, and the nuclear apparatus. **(E)** Dorsal view of a specimen, showing the bristles of dorsal kineties. **(F–H)** Surface view **(F)** and optical sections **(G,H)** of the 2-week-old resting cyst; arrowheads in **(F,G)** mark the hyaline ridges, and opposed arrowheads in **(H)** mark the thick cell wall. Note the separate macronuclear nodules in **(H)**. AZM, adoral zone of membranelles; C, crystals; CV, contractile vacuole; DK1, dorsal kinety 1; DM, dorsomarginal row; FV, food vacuoles; L, lipid droplets; MA, macronucleus; MI, micronucleus; S, scutum; TC, transverse cirri; UM, undulating membranes. Bars: 30 μm **(A–E)**; 15 μm **(F–H)**.

**FIGURE 2:**
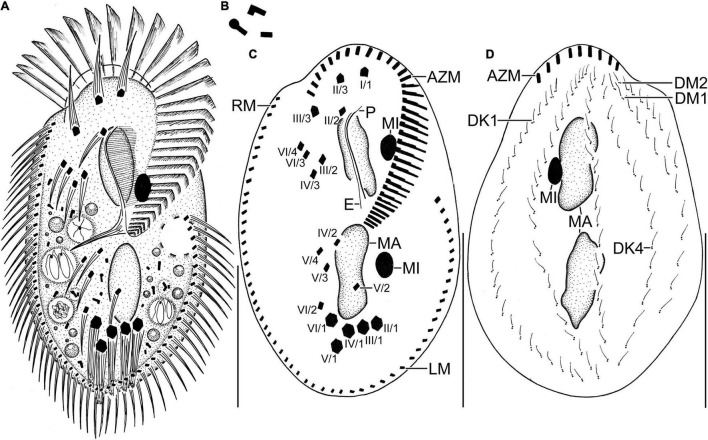
Line diagrams of *Histriculus tolerans* n. sp. from life **(A,B)** and after protargol impregnation **(C,D)**. **(A)** Ventral view of a representative specimen, length 70 μm. **(B)** Crystals. **(C)** Ventral view of the holotype specimen, showing the infraciliature and nuclear apparatus. **(D)** Dorsal view of a paratype specimen, showing the dorsal kineties, dorsomarginal rows, and nuclear apparatus. AZM, adoral zone of membranelles; DK1, 4, dorsal kineties; DM1, 2, dorsomarginal rows; E, endoral; LM, left marginal row; MA, macronucleus; MI, micronucleus; P, paroral; RM, right marginal row. Numbering of cirri according to Wallengren (1900). Bars: 30 μm.

**FIGURE 3:**
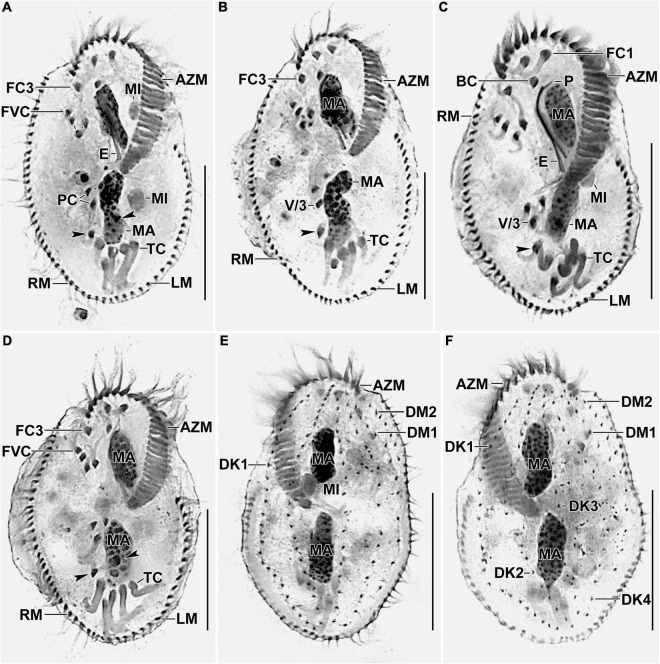
Photomicrographs of *Histriculus tolerans* n. sp. after protargol impregnation. **(A–D)** Ventral view of the holotype **(A)** and paratype specimens **(B–D)**, showing the infraciliature and nuclear apparatus. Arrowheads in **(A–D)** mark the pretransverse ventral cirri. **(E,F)** Dorsal view of the paratype specimens. AZM, adoral zone of membranelles; BC, buccal cirrus; DK1–4, dorsal kineties; DM1, 2, dorsomarginal rows; E, endoral; FC1–3, frontal cirri; FVC, frontoventral cirri; LM, left marginal row; MA, macronuclear nodules; MI, micronuclei; P, paroral; PC, postoral ventral cirri; PTC, pretransverse ventral cirri; RM, right marginal row; TC, transverse cirri; V/3, posteriormost postoral ventral cirri. Bars: 30 μm.

**FIGURE 4:**
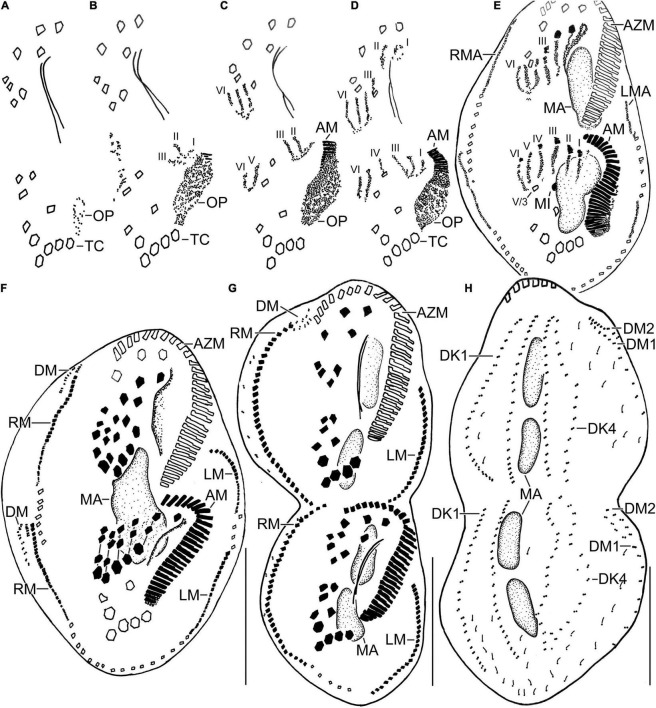
Line diagrams of dividers of *Histriculus tolerans* n. sp. after protargol impregnation. **(A)** Oral primordium develops close to the leftmost transverse cirri (II/1). **(B)** Basal bodies extend towards the anterior right of the oral primordium, forming opisthe anlagen I–III. Cirrus V/4 disaggregates and forms anlagen V and VI for the opisthe; whether this anterior portion of this anlagen contributes to the formation of proter anlagen was not clear from the stages recorded. **(C)** Cirrus VI/3 disaggregates to form anlagen IV–VI for the proter. **(D)** Cirrus II/2 and III/2 disaggregate and form the anlagen II and III of the proter; cirrus IV/2 disaggregates and forms the opithe anlagen III. **(E–G)** Six anlagen are produced both for the proter and opisthe that segregate in 1:3:3:3:4:4 pattern (connecting lines in **F**), forming typical 18 frontal–ventral–transverse cirri. Marginal anlagen forms within row, utilizing 1–3 parental cirri. Anlagen for dorsomarginal rows originates close to the anterior end of newly formed right marginal rows. **(H)** Anlagen for dorsal kineties are formed within row; dorsal kinety 3 undergoes simple fragmentation to form kineties 3 and 4. Dorsomarginal rows originate close to the anterior end of the right marginal row anlagen and shift to the dorsal surface in late dividers. AM, adoral membranelles; AZM, adoral zone of membranelles; DK1, 4, dorsal kineties; DM1, 2, dorsomarginal rows; LM, left marginal row; LMA, left marginal anlagen; MA, macronuclear nodules; MI, micronuclei; RM, right marginal row; RMA, right marginal anlagen; TC, transverse cirri; V/3, posteriormost postoral ventral cirri. Numerals denote cirral anlagen. Bars: 30 μm.

**FIGURE 5:**
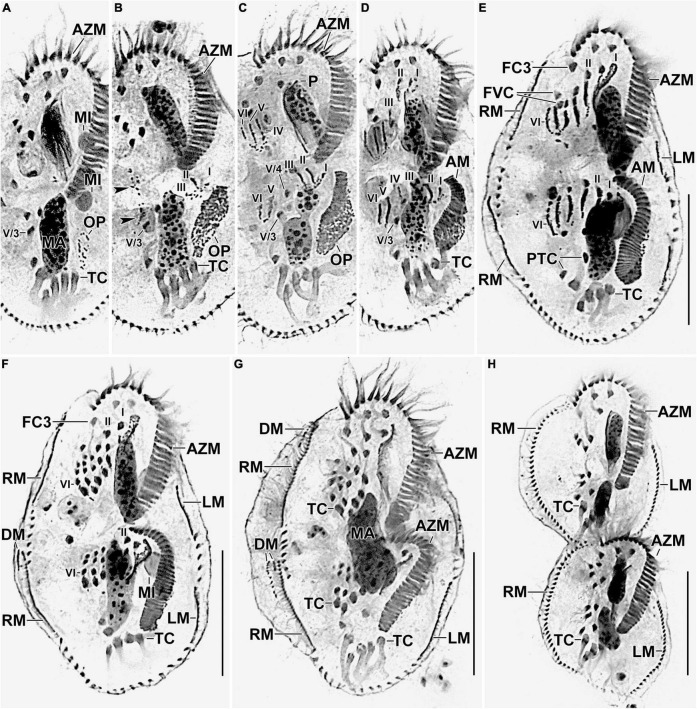
Photomicrographs of *Histriculus tolerans* n. sp. after protargol impregnation. **(A)** Oral primordium develops close to the leftmost transverse cirri (II/1). **(B)** Basal bodies extend toward the anterior right of the oral primordium, forming opisthe anlagen I–III. Arrowheads mark the disaggregation of cirrus V/4 that forms anlagen V and VI for the opisthe in later stages. **(C–H)** For details on the stages, refer to the legend of Figure 4. AM, adoral membranelles; AZM, adoral zone of membranelles; DM, dorsomarginal rows; FC1–3, frontal cirri; FVC, frontoventral cirri; LM, left marginal row; LMA, left marginal anlagen; MA, macronuclear nodules; MI, micronuclei; PTC, pretransverse ventral cirri; RM, right marginal row; RMA, right marginal anlagen; TC, transverse cirri; V/3, posteriormost postoral ventral cirri. Numerals denote cirral anlagen. Bars: 30 μm.

**FIGURE 6:**
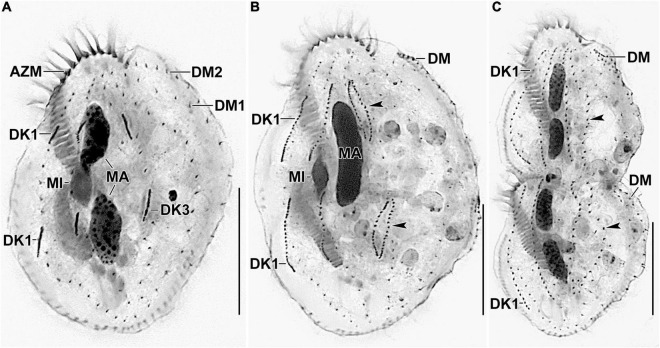
Photomicrographs of *Histriculus tolerans* n. sp. after protargol impregnation. **(A)** Anlagen for dorsal kineties 1–3 are formed within row. **(B,C)** Arrowheads point to the dorsal kinety 3 undergoing simple fragmentation to form kineties 3 and 4. Dorsomarginal rows originate close to the right marginal rows and shift to the dorsal surface in late dividers. AZM, adoral zone of membranelles; DK1, dorsal kinety 1; DM1, 2, dorsomarginal rows; MA, macronuclear nodules; MI, micronuclei. Bars: 30 μm.

*Histriculus tolerans* n. sp. (Figures 1–6 and Table 1).

**TABLE 1:** Morphometric data on *Histriculus tolerans* n. sp.

**Characteristic^a^**	**Mean**	***M***	***SD***	***SE***	**CV**	**Min**	**Max**	***n***
Body, length	61.3	61.0	3.3	0.7	5.4	55.0	66.0	25
Body, width	41.5	42.0	4.0	0.8	9.7	35.0	47.0	25
Body length/width, ratio	1.5	1.5	0.1	0.0	7.6	1.3	1.7	25
Body width/length, ratio	0.7	0.7	0.1	0.0	9.1	0.6	0.8	25
Anterior body end to proximal end of adoral zone, distance	31.3	31.0	1.4	0.3	4.6	27.0	34.0	25
Body length/AZM length, ratio	2.0	2.0	0.1	0.0	7.0	1.7	2.2	25
Anterior body end to proximal end of adoral zone,% of body length	51.2	51.6	3.4	0.7	6.6	45.4	57.9	25
Anterior body end to distal end of adoral zone, distance	7.9	8.0	1.0	0.3	13.2	6.0	9.0	11
DE-value^*b*^	0.3	0.3	0.1	0.0	20.5	0.2	0.3	11
Adoral membranelles, number	31.8	32.0	1.2	0.3	3.7	29.0	33.0	21
Adoral membranelles, width of largest base	5.7	6.0	0.5	0.1	8.2	5.0	6.0	11
Gap between adoral zone of membranelles and anterior of paroral membrane	8.7	9.0	1.1	0.3	12.6	7.0	11.0	11
Anterior body end to paroral membrane, distance	10.4	10.0	0.9	0.3	8.9	9.0	12.0	11
Paroral membrane, length	15.4	15.0	0.9	0.3	6.0	14.0	17.0	11
Anterior body end to endoral membrane, distance	13.8	14.0	1.8	0.5	12.9	11.0	16.0	11
Endoral membrane, length	14.8	14.0	1.2	0.4	8.1	13.0	17.0	11
Anterior body end to anterior macronuclear nodule, distance	11.0	11.0	1.0	0.3	9.1	10.0	13.0	11
Posterior body end to posterior macronuclear nodule, distance	12.7	13.0	1.4	0.4	11.2	10.0	15.0	11
Macronuclear figure, length	38.4	38.0	3.2	1.0	8.3	33.0	45.0	11
Anterior macronuclear nodule, length	15.4	15.0	1.3	0.3	8.5	14.0	19.0	19
Anterior macronuclear nodule, width	6.2	6.0	1.0	0.2	15.6	5.0	8.0	19
Macronuclear nodules, number	2.0	2.0	0.0	0.0	0.0	2.0	2.0	19
Anterior body end to anterior micronucleus, distance	23.3	23.0	6.6	2.0	28.4	13.0	32.0	11
Anterior micronucleus, length	5.7	6.0	0.7	0.2	12.2	4.0	6.5	13
Anterior micronucleus, width	3.5	3.5	0.5	0.1	13.0	3.0	4.0	13
Micronuclei, number	1.3	1.0	0.5	0.1	36.7	1.0	2.0	13
Anterior body end to right marginal row, distance	8.3	9.0	1.3	0.4	16.3	5.0	10.0	11
Right marginal row, number of cirri	34.4	34.0	1.6	0.4	4.7	31.0	37.0	21
Anterior body end to left marginal row, distance	25.6	27.0	2.6	0.8	10.1	21.0	28.0	11
Left marginal row, number of cirri	23.9	24.0	1.9	0.4	7.9	20.0	27.0	21
Gap between last cirri of marginal rows	2.0	2.0	1.2	0.4	59.2	1.0	5.0	11
Frontal cirri, number	3.0	3.0	0.0	0.0	0.0	3.0	3.0	21
Anterior body end to buccal cirrus, distance	9.5	10.0	0.8	0.2	8.6	8.0	10.0	11
Buccal cirrus, number	1.0	1.0	0.0	0.0	0.0	1.0	1.0	21
Anterior body end to posteriormost frontoventral cirrus, distance	20.3	20.0	0.8	0.2	3.9	19.0	21.0	11
Frontoventral cirri, number	4.0	4.0	0.0	0.0	0.0	4.0	4.0	21
Anterior body end to posteriormost postoral ventral cirrus, distance	38.5	38.0	1.7	0.5	4.4	36.0	41.0	11
Postoral ventral cirri, number	3.0	3.0	0.0	0.0	0.0	3.0	3.0	21
Posterior body end to anterior pretransverse ventral cirrus, distance	18.2	18.0	1.3	0.4	7.3	15.0	20.0	11
Posterior body end to rear pretransverse ventral cirrus, distance	14.9	15.0	1.4	0.4	9.2	11.0	16.0	11
Pretransverse ventral cirri, number	2.0	2.0	0.0	0.0	0.0	2.0	2.0	21
Posterior body end to rear transverse cirrus, distance	7.8	8.0	0.9	0.3	11.2	6.0	9.0	11
Transverse cirri, number	5.0	5.0	0.0	0.0	0.0	5.0	5.0	21
Dorsal kineties, number	6.0	6.0	0.0	0.0	0.0	6.0	6.0	15
Anterior body end to dorsal kinety 1, distance	6.7	7.0	1.0	0.3	15.0	5.0	8.0	11
Dorsal kinety 1, number of bristles	25.8	26.0	1.7	0.4	6.4	23.0	28.0	15
Anterior body end to dorsal kinety 2, distance	4.7	5.0	1.0	0.3	21.3	3.0	6.0	11
Dorsal kinety 2, number of bristles	23.3	23.0	1.3	0.3	5.8	21.0	25.0	15
Anterior body end to dorsal kinety 3, distance	5.4	5.0	0.7	0.2	12.6	4.0	6.0	11
Dorsal kinety 3, number of bristles	18.7	19.0	2.0	0.5	10.6	16.0	23.0	15
Anterior body end to dorsal kinety 4, distance	6.2	6.0	1.0	0.3	15.9	5.0	8.0	11
Dorsal kinety 4, number of bristles	16.5	17.0	0.9	0.2	5.5	15.0	18.0	15
Dorsomarginal row 1, number of bristles	11.9	12.0	0.9	0.2	7.4	11.0	14.0	15
Dorsomarginal row 2, number of bristles	8.3	9.0	1.4	0.4	17.4	6.0	11.0	15

*CV, coefficient of variation (%); M, median; Max, maximum; Mean, arithmetic mean; Min, minimum; n, number of individuals investigated; SD, standard deviation; SE, standard error of arithmetic mean.*

*^a^Data based on mounted, protargol-impregnated, and randomly selected specimens from several clonal cultures fed with Chlorogonium elongatum (measurements in micrometer, μm).*

*^b^Distal end of the adoral zone (Berger, 2006).*

#### Diagnosis

Body size about 70 × 40 μm *in vivo*. Body outline elliptical. Two elongate ellipsoidal macronuclear nodules and one or two micronuclei. Cytoplasm colorless. Buccal cirrus at the anterior end of undulating membranes. Adoral zone about 51% of body length, with 32 membranelles on average. About 34 cirri in the right and 24 cirri in the left marginal row. Six dorsal kineties with 26 bristles in kinety 1.

#### Type Locality and Habitat

Water sample was collected from a small stream having polluted water from the outlet of an industrial company in Onsan, Ulsan, South Korea (35°25′55.9′′ N 129°21′07.2′′ E). At the time of collection, the water temperature was 15°C, pH 7.2, and salinity (psu) 0 (freshwater).

#### Type Material

A protargol slide with the holotype specimen (Figures 2C, 3A) circled in black ink is deposited at the National Institute of Biological Resources, Incheon, South Korea, with registration number NIBRPR0000111041. Two paratype slides are also deposited with registration numbers NIBRPR0000111039 and NIBRPR0000111040.

#### Etymology

The species group name *tolerans* (tolerating) refers to its ability to tolerate heavy metals present at the sampling site, i.e., outlet of the industrial waste.

### Morphological Description

Size *in vivo* 60–80 × 30–45 μm, usually about 70 × 40 μm, as calculated from some *in vivo* measurements (*n* = 7) and the morphometric data in Table 1, adding 15% for preparation shrinkage (Kumar et al., 2016), on average 60 × 40 μm in protargol preparations. Body rigid, outline elliptical with round anterior and posterior body end, dorsoventrally flattened about 2:1 (Figures 1A–E, 2A, 3A–D and Table 1). Nuclear apparatus in central quarters of cell in or slightly left of midline, composed of two macronuclear nodules and one or two micronuclei (Figures 1A,B,E, 2A–C, 3A–F and Table 1). Macronuclear nodules narrowly ellipsoidal to ellipsoidal, on average 15 × 6 μm in protargol preparations; contain many small nucleoli, 2–4 μm across. Micronuclei near or attached to macronuclear nodules, ellipsoidal, on average 5.7 × 3.5 μm in protargol preparations. Contractile vacuole in mid-body near left cell margin (Figures 1B,E, 2A). Cortical granules absent. Cytoplasm colorless, filled with cytoplasmic granules, fat droplets, and crystals of usual shapes (Figures 1B–D, 2A,B). Food vacuoles mainly in the posterior half of cell, *in vivo* up to 15 μm across, contains bacteria and flagellates (*Chlorogonium elongatum*) (Figures 1C, 2A). Movement with rapid crawling over and between soil particles.

Cirral pattern stylonychid (Berger, 1999). Invariably three, slightly enlarged, *in vivo* about 14-μm long frontal cirri, right cirrus posterior to distal end of adoral zone membranelles, left cirrus anterior of distal end of undulating membranes (Figures 2A,B, 3A–D). Buccal cirrus, about 10 μm distant from the anterior body end in protargol preparations, right of anterior end of undulating membranes (Figures 2A,B, 3A–D). Four frontoventral cirri, arranged in opposed tick mark shape (Figures 2A,B, 3A–D). Three postoral ventral cirri behind the buccal vertex and two slightly obliquely arranged pretransverse ventral cirri. Invariably, five distinctly enlarged transverse cirri, *in vivo* about 14 μm long, four leftmost reached up to the posterior body margin. Marginal rows confluent posteriorly, cirri about 12 μm long in protargol preparation. Gap between last cirri of rows about 2 μm in protargol preparation, left row composed of an average of 24 cirri; right row of 34 cirri (Figures 2A,B, 3A–D and Table 1).

Invariably six dorsal kineties, including two dorsomarginal rows, with bristles length at about 4 μm *in vivo* and about 2–3 μm in protargol preparations (Figures 1E, 2D, 3E,F). Kinety 1 slightly shortened anteriorly, consists of 23–28 bristles; kinety 2–4 bipolar, with 21–25, 16–23, and 15–18 bristles, respectively. Dorsomarginal row 1 shortened posteriorly, i.e., reaching two-third of body length, consists of 11–14 bristles; row 2 distinctly shortened posteriorly, with 6–11 bristles (Figures 1E, 2D, 3E,F, and Table 1).

Adoral zone about 51% of body length, composed of, on average, 32 membranelles, with cilia about 15 μm long *in vivo*. Paroral and endoral membranes of about equal length, slightly curved and nearly parallel (Figures 1C,D, 2A,C, 3A–D). Paroral commences about 10 μm posterior of anterior body end; endoral commences about 3 μm posterior of anterior end of paroral, at the level of buccal cirrus (Figures 1C,D, 2A,C, 3A–D and Table 1). Pharyngeal fibers of ordinary length and structure extend obliquely toward the right body margin (Figure 2A).

#### Resting Cyst

Two-week-old resting cysts about 40 μm in diameter *in vivo*; cyst surface with hyaline ridges, about 1.5–2.5 μm high (Figures 1F–H). Cyst wall about 1.5 μm thick. Cyst content attached to the wall, composed of lipid droplets and separate macronuclear nodules (Figures 1F–H).

#### Divisional Morphogenesis

Divisional morphogenesis resembles the type species *H. histrio*, except that the oral primordium does not contribute to the anlage II of the proter (for a review, see Berger, 1999; Gupta et al., 2006). The parental adoral zone is retained unchanged for the proter, while that of opisthe is formed from the oral primordium that originates close to the transverse cirrus II/1 (Figures 4A–G, 5A–H). Five parental cirri (II/2, III/2, IV/2, IV/3, and V/4) and the parental undulating membranes are involved in the formation of six anlagen each for proter and opisthe. The proter anlage I generate from partial reorganization of the paroral and endoral, anlage II from disaggregation of cirrus II/2, anlage III from cirrus III/2, and anlagen IV–VI from cirrus IV/3. The involvement of cirrus V/4 in the formation of proter anlagen IV–VI was not clear in our observation. The opisthe anlagen I–III originate from oral primordium, anlagen IV from cirrus IV/2, and anlagen V and VI from cirrus V/4. The paroral and endoral and the first frontal cirrus I/1 are formed from anlage I. The postoral ventral cirrus V/3 is not involved in anlagen formation. The 18 frontal–ventral–transverse cirri arise from six anlagen by splitting in a 1:3:3:3:4:4 pattern (Figures 4A–G, 5A–H).

The marginal primordia arise at two levels by within-row anlagen formation by utilizing one to three of the parental cirri. The marginal primordia elongate utilizing three to five parental cirri and differentiate into new marginal rows. The remaining parental cirri are resorbed (Figures 4E–G, 5E–H).

On the dorsal surface, three anlagen are formed within row from dorsal kineties 1, 2, and 3 at two levels (one set each for the proter and the opisthe). The third dorsal anlage fragments in the middle and gives rise to the third and fourth dorsal kineties of equal length. The two dorsomarginal rows arise near the anterior end of two newly formed right marginal anlagen and move from the lateral to the dorsal surface (Figures 4H, 6A–C).

The nuclear division proceeds as usual, i.e., the macronuclear nodules fuse to form a single mass in middle dividers, which divides two times to produce the typical four nodules in late dividers. The micronuclei undergo mitotic division as usual (Figures 4E–H, 5E–H, 6A–C).

#### Occurrence and Ecology

As yet found only at the type locality, i.e., heavy metal-contaminated water from an industrial outlet in Onsan, Korea. The main physico-chemical parameters during the sampling month, i.e., April 2016, were the following: water temperature, 15°C; pH, 7.2; DO, 9.9 mg/L; BOD, 5.4 mg/L; COD, 8.7 mg/L; SS, 12.5 mg/L; T-N, 9.1 mg/L; T-P, 0.27 mg/L; TOC, 3.6 mg/L; EC, 813 μS/cm. The heavy metal concentrations were the following: Cd, 0.0132 mg/L; Pb, 0.0117 mg/L; As, 0.0808 mg/L; Hg, 0.0038 mg/L; Cu, 0.6945 mg/L; Zn, 3.3035 mg/L; Cr, 0.0674; and Ni 0.1513 mg/L. The concentrations of a nearby less-contaminated site were Cd, less than the limit of quantification (LLQ); Pb, LLQ; As, LLQ; Hg, LLQ; Cu, 0.0495 mg/L; Zn, 0.1230 mg/L; Cr, 0.0222 mg/L; and Ni, 0.0167 mg/L. The heavy metal concentrations in most non-contaminated sites have not been measured so far. However, the concentrations of heavy metals in some of the measured ones were mostly LLQ. The coliform count was 11,000/100 mL (data retrieved from the water quality monitoring system of the Korean Ministry of Environment^3^).

#### SSU rRNA Gene Sequence and Phylogeny

**FIGURE 7:**
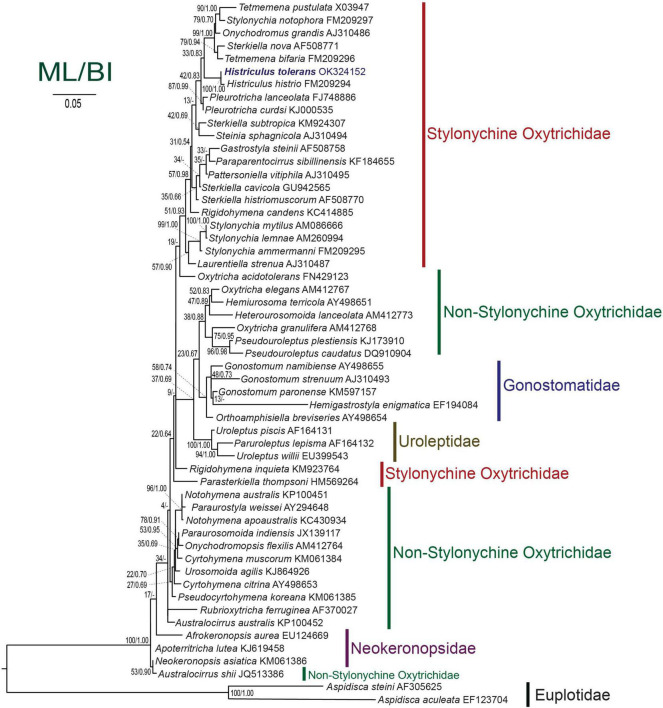
Maximum likelihood tree inferred from the small subunit rRNA gene sequences showing the position of *Histriculus tolerans* n. sp. (bold). Codes after the species names are GenBank accession numbers. Numbers at the nodes represent the bootstrap values of maximum likelihood and the posterior probability of bayesian analysis. A hyphen (-) represents minor differences between the bayesian and maximum likelihood tree topologies. The scale bar corresponds to five substitutions per 100 nucleotide positions.

The SSU rRNA gene sequence of *H. tolerans* is 1,663 bp in length and has a GC content of 44.5%. Phylogenetic analyses inferred from the SSU rRNA gene sequences using ML and BI present similar topologies; therefore, only the ML tree is shown here (Figure 7). Phylogenetic trees consistently place the new species within the stylonychine oxytrichids, clustering in a clade with *H. histrio*.

## Discussion

### Comparison With Related Species

The genus *Histriculus* contains six species that should be compared with *H. tolerans*, namely, *H. histrio*, *Histriculus sphagni* (Stokes, 1891) Corliss, 1960; *Histriculus complanatus* (Stokes, 1887) Corliss, 1960; *Histriculus minimus* (Groličre, 1975) Berger and Foissner, 1997; *H. vorax* (Stokes, 1891) Corliss, 1960; and *Histriculus similis* (Quennerstedt, 1867) Corliss, 1960. Except for the type species, i.e., *H. histrio*, the description of the other species is mainly based on live observations, and thus detailed reinvestigations based on standard methods, especially live observations and protargol impregnation, were recommended by Berger (1999).

The type species can be easily separated from *H. tolerans* by having a larger body size *in vivo* (over 100 μm vs. about 70 μm), more adoral membranelles (40–58 vs. 29–33), and cirri in left (23–35 vs. 20–27) and right (33–51 vs. 31–37) marginal rows, bristles in dorsal kinety 1 (50–58 vs. 23–28), kinety 2 (38–46 vs. 21–25), kinety 3 (33–42 vs. 16–23), and dorsomarginal row 1 (25–35 vs. 11–14) (average values from Berger, 1999; Gupta et al., 2006; Kwon and Shin, 2011).

*H. sphagni* can be distinguished from *H. tolerans* in having rather straight (vs. slightly curved) undulating membranelles, additional cirrus (vs. no such cirrus) behind the frontal cirrus, and five (vs. six) dorsal kineties (Berger, 1999).

The new species can be separated from *H. complanatus* in the arrangement of transverse cirri, i.e., five transverse cirri arranged in a more or less tick mark shape (vs. arranged in rows) of which four leftmost (vs. three rightmost) reaching up to the cell margin (vs. protrudes beyond the posterior end) of the cell, buccal cirrus at the anterior right (vs. distinctly posterior) of undulating membranes, and marginal cirri of the same length [vs. increase in length posteriorly; for which Berger (1999) mentioned that inconspicuous caudal cirri could be present] throughout the body (data from illustrations of Stokes, 1887; Vuxanovici, 1961).

*H. minimus* differs from *H. tolerans* by a smaller body size (30–40 vs. 60–75 μm), arrangement of micronuclei, i.e., one in between two macronuclear nodules (vs. one or two attached at various positions or near to the nodules), and 18–21 (vs. 31–37) cirri in the right marginal row (Berger, 1999).

*H. vorax* has been reported with rather variable shapes, i.e., about 170 μm (Stokes, 1891), small adult cells 70–140 × 50–60 μm, and large adult cells 190–250 × 100–120 μm (Curds, 1966). The body margin is often indented, with the left margin concave and the right convex, which was not observed in the present species. The new species can be distinguished from *H. vorax* in having a smaller body length, i.e., 60–75 × 40–55 μm. Further differences include widely spaced and fewer marginal cirri (vs. narrowly spaced with 31–37 cirri in the right marginal row) in *H. vorax*. Berger (1999) mentioned the possibility that *H. vorax* may possess caudal cirri due to the longer and prominently projecting cirri beyond the posterior end of the cell.

*H. similis* can be distinguished from *H. tolerans* in having a long body *in vivo*, i.e., 110–130 μm (vs. 60–75 μm), body about three (vs. two) times as long as broad, and the adoral zone of membranelles was about 38% (vs. 51%) of body length.

The divisional morphogenesis of *H. tolerans* resembles that of *H. histrio* as described by Gupta et al. (2006). The only significant difference observed was that the oral primordium in *H. tolerans* does not contribute to the formation of anlage II of the proter (for a review, see Berger, 1999; Gupta et al., 2006). For the remaining *Histriculus* species, no morphogenetic data are known. However, Curds (1965, 1966) reported morphogenesis in *H. vorax* by the formation of an endogenous bud that also needs to be reinvestigated since Pang and Zhang (1981) assumed it to be the cannibalism induced by overfeeding. No endogenous bud formation was observed in *H. tolerans*.

### Phylogenetic Analyses

The SSU rRNA gene sequence of *H. tolerans* matches well with *H. histrio* (99% similarity and four base pair difference), the only species within the genus for which gene sequence is available. In the phylogenetic analyses, the new species clustered with the type species with the full support of 100/1.00 (ML/BI). The detailed analyses are restricted due to the limited sequences available within the genus; however, *Histriculus* is well fitted in the subfamily Stylonychinae clustering in a clade with *Tetmemena*, *Stylonychia*, and *Sterkiella* species (Figure 7).

In our analyses, the genus *Sterkiella* appeared to be a polyphyletic group, possibly due to the differences in the morphogenetic pattern (Kumar et al., 2015, 2017; Bharti et al., 2018). Similarly, a slight variation in morphogenesis was observed in the present study, i.e., no contribution of the oral primordium toward the formation of anlage II of the proter in contrast to the type species where such contribution was reported by Gupta et al. (2006). Furthermore, recent observations on the resting cyst structures have resulted in the identification of the cryptic ciliate species (Foissner, 2016; Kumar et al., 2016; Bharti et al., 2019). We believe that the detailed observations, i.e., future addition of cyst data along with morphology and morphogenesis, as well as molecular sequences, for the remaining *Histriculus* species will clarify whether the genus *Histriculus* stays monophyletic or polyphyletic.

### Heavy Metal Toxicity in Ciliates

Ciliates have been reported to play a significant role in the bioaccumulation or bio-concentration since they come directly in contact with chemicals in contrast to other single-cell microbes, such as bacteria, yeast, and algae, that possess a cell wall and may affect the uptake of chemicals (Cooley et al., 1972; Carter and Cameron, 1973). Furthermore, several studies have highlighted the potential of protozoan ciliates as a model organism for various *in vitro* metal toxicological assessments as well as their role in the self-purification of natural aquatic ecosystems (Cooley et al., 1972; Carter and Cameron, 1973; Larsen et al., 1997; Pauli and Berger, 1997; Sauvant et al., 1999; Bogaerts et al., 2001; Schultz et al., 2005; Vilas-Boas et al., 2020).

The heavy metal concentration during the year (2015–2018) from the sampling site ranges significantly and on many occasions rose higher than the permissible levels, e.g., Cd concentration reached up to 0.252 mg/L, Pb up to 0.44 mg/L, and As up to 0.456 mg/L (data retrieved from the water quality monitoring system of the Korean Ministry of Environment, see text footnote 3). Thus, it is believed that the species present in this habitat must possess some defense mechanism to overcome the stress of the heavy metal. The present study reports a novel species that might have the potential to survive heavy metal stress and that it can be used as a model organism for evaluating heavy metal toxicity. However, further studies are required for generating reference data that can be used in future ecotoxicological analyses using *H. tolerans*.

## Data Availability Statement

The original contributions presented in the study are publicly available. This data can be found here: National Center for Biotechnology Information (NCBI) BioProject database under accession number OK324152.

## Author Contributions

MS and SK collected the samples. SK and DB performed all the experiments (permanent preparations, illustrations, photomicrographs, etc.) and identified the species and wrote the first draft. SS and SK performed the phylogenetic analyses. All authors contributed to the writing of the final manuscript.

## Conflict of Interest

The authors declare that the research was conducted in the absence of any commercial or financial relationships that could be construed as a potential conflict of interest.

## Publisher’s Note

All claims expressed in this article are solely those of the authors and do not necessarily represent those of their affiliated organizations, or those of the publisher, the editors and the reviewers. Any product that may be evaluated in this article, or claim that may be made by its manufacturer, is not guaranteed or endorsed by the publisher.

## Footnotes

1
https://mafft.cbrc.jp/alignment/server/

2
http://tree.bio.ed.ac.uk/software/figtree/

3
http://water.nier.go.kr
